# Understanding what factors influence community health worker involvement in hypertension service delivery in Kenya: applying a community health system lens

**DOI:** 10.1093/heapol/czag020

**Published:** 2026-02-24

**Authors:** Nancy Kagwanja, Robinson Oyando, Syreen Hassan, Brahima A Diallo, Jainaba Badjie, Ruth Lucinde, Noni Mumba, Sam Kinyanjui, Pablo Perel, Anthony Etyang, Nadia Aaliyan, Hassan Leli, Ellen Nolte, Benjamin Tsofa

**Affiliations:** Health Systems and Research Ethics Department, KEMRI-Wellcome Trust Research Programme, P.O BOX 230-80108, Kilifi, Kenya; Health Economics Research Unit, KEMRI Wellcome Trust Research Programme, P.O BOX 230-80108, 197 Lenana Place, Nairobi, Kenya; Department of Health Service Research and Policy, London School of Hygiene and Tropical Medicine Faculty of Public Health and Policy, Keppel Street, London WC1E 7HT, United Kingdom; Nutrition and Planetary Health, MRC Unit the Gambia at LSHTM, Atlantic Boulevard, P.O BOX 273, Fajara, Banjul, The Gambia; Nutrition and Planetary Health, MRC Unit the Gambia at LSHTM, Atlantic Boulevard, P.O BOX 273, Fajara, Banjul, The Gambia; Department of Epidemiology and Demography, KEMRI-Wellcome Trust Research Program, P.O BOX 230-80108, Kilifi, Kenya; Health Systems and Research Ethics Department, KEMRI-Wellcome Trust Research Programme, P.O BOX 230-80108, Kilifi, Kenya; Training Department, KEMRI-Wellcome Trust Research Program, P.O BOX 230-80108, Kilifi, Kenya; Nuffield Department of Medicine, Oxford University, Old Road Campus, Oxford, OX3 7BN, United Kingdom; Department of Biochemistry, Pwani University, P.O BOX 195-80108, Kilifi, Kenya; School of Business Studies, Strathmore University, P.O BOX 59857-00200, City Square, Nairobi, Kenya; Department of Non-Communicable Disease Epidemiology, London School of Hygiene and Tropical Medicine, Keppel Street, London, WC1E 7HT, United Kingdom; Department of Epidemiology and Demography, KEMRI-Wellcome Trust Research Program, P.O BOX 230-80108, Kilifi, Kenya; Kilifi County Department of Health, P.O BOX 9, 80108, Kilifi, Kenya; Kilifi County Department of Health, P.O BOX 9, 80108, Kilifi, Kenya; Department of Health Service Research and Policy, London School of Hygiene and Tropical Medicine Faculty of Public Health and Policy, Keppel Street, London WC1E 7HT, United Kingdom; Health Systems and Research Ethics Department, KEMRI-Wellcome Trust Research Programme, P.O BOX 230-80108, Kilifi, Kenya; School of Business Studies, Strathmore University, P.O BOX 59857-00200, City Square, Nairobi, Kenya

**Keywords:** community, health system, capacity, hypertension, community health worker, health policy

## Abstract

The systematic involvement of community health workers (CHWs) in hypertension management can improve outcomes and achieve blood pressure control. However, much of this evidence is from effectiveness trials conducted under ideal conditions, with little evidence from programmes operating in routine conditions. In Kenya, recent policy changes have expanded CHW roles to routinely incorporate non-communicable disease (including hypertension) service delivery. We undertook an exploratory descriptive qualitative study in one county, examining what CHWs now referred to as community health promoters (CHPs) do in relation to hypertension service delivery, influences on their involvement, and considerations for sustainability. We found ad hoc and fragmented CHP involvement in practice despite policy guidance for community-level hypertension service delivery. Drawing on the extended health systems building blocks framework, we identified multiple capacities that can support expanded CHP roles in hypertension care, including the pre-existing community health service structure and societal partnerships, as well as their level of motivation. Policy provisions for CHP professionalization (payment of stipends, provision of CHP kits with varied commodities and training) create an enabling environment. However, sustained adoption of the new CHP roles may be impeded by (i) challenges in meeting the financial and supply chain obligations for stipend payments and commodities, respectively; and (ii) inadequate sensitization of communities and frontline-providers concerning expanded CHP roles and implications for facility-level hypertension care. To effectively implement recent policies, strengthening coordination and communication across all community and health system actors is needed, as well as clarity and deliberation on long-term financing for the community health system.

Key messages

- Recent legislation, policy releases, and an event to launch supplies for health provision at community level have paved the way for formal recognition of community health workers [or community health promoters (CHPs)] in Kenya within the health system, creating a positive policy environment for community health worker involvement in hypertension service delivery in Kenya.
- Existing community health system capacities that could support an expanded role for CHPs in hypertension service delivery include high coverage of motivated CHPs, existing relationships with community members, and partnerships to support household production of health.
- Despite policy directives encouraging hypertension service delivery at the community-level, CHP involvement in hypertension service delivery continues to be ad hoc and fragmented. Other constraints to delivery of the service include fragile linkage between CHPs and health care providers, community members’ scepticism about CHP readiness to deliver hypertension care, and inadequate financing and planning for the expansion of CHP roles.
- Rolling out sustainable involvement of CHPs in hypertension care will require wider system changes around financing, investment in human resources, commodities at both community and broader health system level, and community confidence in CHPs to deliver this role.

## Introduction

People living in low- and middle-income countries are disproportionately affected by the rising burden of hypertension, accounting for about three-quarters of the adult population living with this condition (WHO 2023). The prevalence of hypertension is high in both rural and urban settings, but disease awareness, treatment, and control are lower in rural areas (Ataklte et al. 2015, Nulu et al. 2016, Bosu et al. 2019). For example, in Kenya, the proportion of people living in rural areas who were aware of their hypertension was 12.3% compared to 20% in urban areas (Mohamed et al. 2018).

Reasons for poor outcomes along the hypertension care cascade are multifaceted. They include individual-level factors such as treatment-seeking practices (Gideon et al. 2020, Herbst et al. 2021); service-level factors such as patient–provider interactions; system-wide factors, e.g. availability of drugs in health facilities and long-waiting times (Vedanthan and Fuster 2011, Attaei et al. 2017, Oyando et al. 2023a, Sanju et al. 2023); and broader socio-economic factors such as costs related to travel and purchasing of drugs (Attaei et al. 2017, Oyando et al. 2023b). Interventions seeking to improve outcomes for people living with hypertension therefore need to be multidimensional. One such intervention includes involving community health workers (CHWs) to support patient-centred care (WHO 2023). Evidence from South-East Asia and Latin America suggests that the systematic involvement of CHWs in hypertension management can achieve reductions in cardiovascular disease risk and improve blood pressure (BP) control (Schwalm et al. 2019, Jafar et al. 2020). In Kenya, community-centred interventions involving CHWs were shown to improve linkage to care and BP control (Pastakia et al. 2017, Vedanthan et al. 2021). Yet, important evidence gaps remain. For example, there is little literature about the routinization, embeddedness and sustainability of interventions involving CHWs in local health systems beyond research trial periods. This study sought to contribute to filling this evidence gap.

In this paper, we define CHWs as lay workers without professional health training who provide health services in the community. Community health systems literature suggests that a holistic understanding of CHW programmes in service delivery requires analysis beyond individual CHW attributes (such as their skills and motivations) to include considerations of the systems requirements for CHW-led interventions, and recognition that CHW programmes straddle both primary health care and community systems (McCord et al. 2012, Schneider and Lehmann 2016). This paper is guided by an understanding of a community health system as a ‘comprehensive set of community-based health providers, bounded by local contexts and existing in relationship with households, health system, governance, and other community structures’ (Schneider and Lehmann 2016). We drew on the extended health systems building blocks framework by Sacks et al. (2019) to interpret our study findings. This Sacks et al. (2019) framework expands the World Health Organization (WHO) health system building blocks to include elements such as household production of health, social determinants of health, and community organizations or societal partnerships that are, directly or indirectly, linked to health. The framework also expands service delivery, health workforce, and information flow dimensions across facility and community components to draw attention to the needs of both.

The work presented in this paper is part of Improving Hypertension Control in Sub-Saharan Africa (IHCoR-Africa), a project that aims to improve hypertension management through a sustainable community-centred approach in two rural sites in Kenya and Gambia. IHCoR-Africa has three inter-related objectives: to examine the experiences, needs, and practices at individual, organizational, and system levels to detect, treat, and control hypertension (Diallo et al. 2024); to determine the optimal diagnostic and risk stratification approaches for people with hypertension (Perkins et al. 2023); and to develop and evaluate the feasibility of a community-centred intervention to improve hypertension care. This paper reports findings from Kenya related to the first aim. We explored whether and what hypertension services CHWs [now called community health promoters (CHPs)] offer and how the health system and broader context shape their involvement, and considered the capacity of the community health system to support and sustain CHPs involvement in hypertension service delivery and how this can be improved.

### Context

Kenya operates under a devolved governance system, comprising a national government and 47 semi-autonomous counties. The national government is responsible for health policy, training, and oversight over national tertiary and referral hospital functions, while counties have responsibility for management of health service delivery (GoK 2012, 2017). Counties are financed through a combination of national transfers (equitable share), own-source revenue (local taxes and fees), and donor support (Ministry of Health 2024 ). Counties allocate healthcare funding based on their priorities and strategic plans (Ministry of Health 2024). Over half (55%) of all health facilities are in private ownership (Moturi et al. 2022); but most Kenyans use government-owned public facilities (MoH 2013).

Kenya's public health system is organized into four tiers with six levels: tier 1, community [community health units (CHUs)], tier 2 primary healthcare (PHC) facilities (dispensaries and health centres), tier 3 sub-county and county referral (primary and secondary referral hospitals), and tier 4 national referral (tertiary hospitals) (MoH 2014, 2018). Boxes 1 and 2 further summarise the community health structure and policy context for delivery of hypertension services at community level, respectively, following a review of health policy documents.

Box 1. **Community health services organization and structure (MoH 2020a, 2020b).**

One community health unit serves up to 5000 people (between 500 and 1000 households). The community health service workforce in a community health unit includes: the community health committee (CHC), which is the governing body for the unit; community health assistants (CHAs) or community health officers (CHO), and community health promoters (CHPs). CHPs are members of the community who are selected by community members during barazas (community meetings) and trained by sub-national health managers and facility-level supervisors to provide mainly preventive and promotive health services to defined households. CHPs are supervised by CHAs or CHOs who are based at the primary health care (PHC) facilities. Every community health unit is linked to a health facility, and CHPs can refer community members to these facilities for care. Kenya's Community Health strategy proposes that one CHA/CHO should oversee 10 CHPs who attend to up to 5000 people (500–1000 households) (MoH 2020b).

Box 2. **Policy context for hypertension service delivery at community level (MoH 2014, 2018, 2020a, 2021 , CGOK 2023, GoK 2023, Parliament 2023, CHU4UHC, 2024).**

In Kenya, CHWs have traditionally been involved in the provision of promotive and preventive care for maternal child health, water sanitation and hygiene, and programmes targeting infectious diseases such as HIV/AIDS and tuberculosis (MoH 2014, 2020b). More strategic involvement of CHWs in the control and management of non-communicable diseases (NCDs) was emphasised in the 2018 Health Sector Strategic Plan and the 2021 NCD Strategy Plans, proposing community-level NCD screening and health education as key strategies for addressing the rising NCD burden (MoH 2018, 2021). More recent strategy documents outline specific proposals for how CHWs [referred to as community health promoters (CHPs), from 2023] would be supported to do their work. These include formal recognition of CHPs as part of the health system, provision of CHP kits, training, and payment of a stipend (CGOK 2023, GoK 2023, Parliament 2023) . These initiatives were also accompanied by roll-out of the electronic Community Health Information System (eCHIS), a national community-level digital health platform which led to issuance of smartphones to CHPs (MoH 2020a, CHU4UHC, 2024). The eCHIS areas of functionality include household enrolment, service delivery, client referral, supply chain management, community-based disease surveillance and client messaging (MoH 2020a). The eCHIS is expected to interface with the Kenya Health Information System (KHIS) for health service reporting. In Kilifi County, which is the setting for this study, the 2023 Kilifi County Community Health Services Act includes payment of the national health insurance fund (NHIF) premiums in addition to payment of stipends for CHPs (CGOK 2023). The community health policy and strategy documents also clarify that the community health committee should have oversight over CHPs (MoH 2020a, 2020b). The renewed attention to PHC and plans to expand the role of CHPs in Kenya should therefore provide a positive policy environment for the roll-out of a community-centred intervention for hypertension.

## Materials and methods

### Study setting

This study was conducted in Kilifi County, a rural region in coastal Kenya, characterized by a high hypertension burden (Etyang et al, 2014, 2016). We included health facilities within the Kilifi Health Demographic Surveillance System (Scott et al. 2012) as prior community engagement for a larger study had been done in this area. Hypertension services are decentralized to lower health system levels with many PHC facilities routinely running hypertension clinics.

### Study design and data collection

We adopted an exploratory, descriptive qualitative study design. The study population included patients, CHPs, healthcare workers, and decision-makers. Study participants were purposively selected as respondents based on their roles [i.e., providing hypertension care (healthcare providers); management or oversight of hypertension services (decision-makers at county and national levels); and/or community health service delivery (CHPs, community health assistants (CHAs)] and their use of hypertension services (patients) (Table 1). Healthcare providers and people living with hypertension who were ≥18 years old were recruited across five health facilities (two health centres, two dispensaries and one hospital). Patients were approached during their clinic day and invited to participate in the study at a time and place of their convenience. We sought patients with varying characteristics and experiences of hypertension care, such as sociodemographic characteristics (e.g. age and sex); stage of the hypertension journey (newly diagnosed (≤6 months); treatment duration; those with complications and comorbidities (i.e. hypertension and diabetes); and those who had stopped seeking care (i.e. stopped using their prescribed medication for at least 3 months or attending scheduled clinic appointments for at least 6 months). Participants who had stopped seeking care were recruited through household screening for hypertension as part of a related IHCoR study (Perkins et al. 2023) or with the help of front-line healthcare providers. Recruitment of participants stopped after achieving the pre-defined categories and attaining data saturation (Rahimi and Khatooni 2024).

**Table 1:** Data collection and participant summary.

Data collection method	Details		
Interviews	People living with hypertension, *n* = 24		
	Frontline providers across health centres, dispensaries, and hospital, *n* = 19		
	County and sub-county decision-makers, *n* = 12		
	National level decision-makers, *n* = 5		
	NGO stakeholders, *n* = 3		
Focus group discussions	People living with hypertension across three health facilities. *n* = 5 FGDs, total participants (*n* = 30)		
Stakeholder workshops	Month held and number of participants		
Participant Category/workshop date	May 2024	July 2024	August 2024
Patients and carers	6 (3 carers)		5 (2 carers)
Community health promoters	5		5
Community health assistants	3		3
Facility managers/frontline providers	4		2
Sub-county health managers	6		5
Physicians (level 4 facilities)	2		1
Nutritionist	1		
County health managers	5		5
National level managers/decision-makers	4		2
Kenya Medical Research Institute KEMRI^a^ community representatives		6	
Religious leaders		5	
Community pharmacists		3	
Total	40	14	27

^a^KEMRI, Kenya Medical Research Institute.

Interview and focus group discussion (FGD) data were collected between June and December 2023. Interviews and FGDs with patients explored their experiences with CHPs, whether they had received hypertension care from them, and views about routine CHP involvement in hypertension services. Interviews with CHPs explored their current roles and experiences with providing hypertension care, while interviews with healthcare providers, managers, and decision-makers explored current strategies for delivery of hypertension services, and perspectives on CHP involvement in hypertension service delivery considering physical, resource, and social factors (see online supplementary material S1). N.K. and R.O. collected data with the support of trained community facilitators. Interviews were conducted in Kiswahili and Giriama (patients and CHPs) and English (healthcare providers and decision-makers) at health facilities or offices. Six interviews [with non-governmental organization (NGO) stakeholders and national-level decision-makers] were conducted on-line using Microsoft Teams. Interviews lasted between 30 and 45 min while FGDs lasted an average of 90 min.

Workshops were conducted between May and August 2024 where we presented preliminary study findings to stakeholders, including people living with hypertension and their caregivers, frontline providers, CHPs and health managers and decision-makers to collate additional input on CHP roles in hypertension care from workshop participants (Table 1).

### Data analysis

Interviews and FGD audio recordings were transcribed verbatim and translated into English. The transcripts, together with notes from the stakeholder workshop sessions, were analysed using a thematic analysis approach (Braun and Clarke 2006). N.K., R.O., S.H., and B.A.D. read and re-read the transcripts to familiarize themselves with the data. After independently coding a sample of two transcripts per participant category, the authors met to collaboratively develop a coding framework. These codes were discussed with E.N. and B.T., after which N.K. applied the coding framework to all transcripts and identified patterns and potential themes. Inductive coding was used primarily, but was combined with a deductive approach drawing on the research questions and Sacks et al.’s conceptual framework. Emerging findings were discussed during reflective sessions with the research team. This iterative process underpins the trustworthiness of the analysis.

## Results

The results are presented in two broad sections. First, current CHP involvement in hypertension care is described. Second, findings are presented on existing capacities that can support CHPs’ roles in hypertension care, as well as potential constraints, using the lens of the extended health systems building blocks framework.

### Ad hoc and fragmented involvement of CHPs in hypertension service delivery in practice

As noted above, recent national policy promotes CHP involvement in hypertension care, yet little evidence was found of policy implementation in practice at the time of data collection. Healthcare providers and decision-makers thought that CHP involvement in hypertension care was largely ad hoc. Sometimes, this included community-level hypertension screening during outreach activities or in health facilities to free up healthcare providers to perform clinical tasks. Most CHPs reported little involvement in hypertension service delivery. Indeed, many people living with hypertension reported that they had not received any hypertension services from CHPs, including measurement of BP:

*They [CHPs] go with the nurse to distribute vaccines in the community… they also distribute drugs for elephantiasis. But anything to do with high blood pressure, we have not heard about that, so when somebody has an attack [hypertensive emergency], then they come here [health facility] so they can get treatment and some information* (Focus Group Discussion participant_B001)

Interviews with decision-makers and NGO respondents suggested that support offered to CHPs to provide hypertension services varied. For example, one NGO respondent reported providing CHPs linked to a hospital with BP machines and paying them a stipend to conduct home blood pressure measurements. Other NGO engagements with CHPs for hypertension care included mobilizing communities for screening at health facilities and home visits to people with hypertension who had stopped treatment. It was unclear whether CHPs were remunerated for these activities. Based on our analysis, there was agreement overall that CHPs and their supervisors, CHAs, were only marginally involved in hypertension care and had not received any formal hypertension care training.

CHPs reported challenges relating to remuneration for their overall work, highlighting the low amounts, inconsistencies, and timeliness of payments.

*‘It's a small amount… it's not the same all the time, but we [CHPs] are people with other responsibilities, the women have left their children, their homes. One husband can understand, another one might not understand, he might get upset until you decide to stop, or he says ‘if you don't have something to do just sit at home’, because you go for an outreach you get [kshs] 500… and sometimes you don't get it immediately the work is done; you wait until you forget that you did [the outreach]… you remember when you see the Mpesa [money transfer] message* (Community Health Promoter_A003)

At the time of data collection, none of the CHPs participating in this study had received government stipends as stipulated in the recent policy directives.

### Enablers of CHP involvement in hypertension service delivery

Despite fragmented hypertension services at community level, CHP involvement in hypertension service delivery was widely seen to be acceptable and appropriate. Several participants highlighted potential facilitators that would support CHP delivery of hypertension care, citing the community health service delivery structure with its health workforce comprising CHPs and their supervisors; CHPs and community member relations; and community organizations and societal partnerships that supported household production of health. These enablers are discussed in turn.

#### Well-functioning community health service delivery structure and motivated CHPs

Many study participants felt that the current organization and functioning of the community health service structure would fit well with the inclusion of CHPs in hypertension care. County and sub-county health managers thought that there was good coverage (90%) of community health units. In these community health units, many of the CHPs had received basic community health training and actively and consistently conducted home visits. All interviewed CHPs reported receiving adequate support from their supervisors (CHAs). This supervision included monthly meetings where CHPs shared feedback from household visits and discussed challenges encountered in the community. CHPs reported calling their supervisors for guidance while conducting household visits. Sometimes, the CHAs also accompanied them to homesteads they perceived to be difficult.

Overall, CHPs appeared motivated to take on additional roles related to hypertension care. Their supervisors (CHAs) also noted that CHPs could fit these roles into their existing workloads and schedules. For example, CHAs thought that hypertension messaging could easily be incorporated into other health education during CHPs’ regular household visits.

‘*To me I feel [involving CHPs in hypertension care] is a good approach… In their activities we encourage them to ask the elder population 60 years and above to come for health checkups, so in the same way we can ask them to give messages about these lifestyle diseases when they visit households* (Community Health Assistant A001)

At the same time, CHPs recognised that conducting home BP measurements would increase the time spent in households, which could reduce their own time to engage in income-generating activities. They however expected that the stipend announced by the government would support CHPs’ active engagement:

*The President stated and we clearly heard him, we have hope, because of his campaign slogan, ‘bottom up’, and because we are on the bottom, we know things are going to change. So, we hold on to that even though we haven't got anything, but it was said that when the budget is out, we will be able to get it [a stipend], we saw him emphasizing and he said there is no volunteering again… just promoters.* (Community Health Promoter_B001)

CHPs also noted that involvement in hypertension care provided an opportunity to expand their scope of practice and potentially increase their legitimacy. The latter appeared to be particularly important to CHPs, as they linked additional roles to training and formal recognition by the health system.

*I would just like that [hypertension] training so that we are also known that we have studied and we have the certification… that will increase our confidence as well… and even if someone was a bit scornful [of CHPs], he will know this one has gone through things, so I see it would improve that integration* (Community Health Promoter_A002)

CHPs felt confident that with appropriate training they could screen, refer, and monitor hypertensive patients. A small number of CHPs also felt confident that they could deliver drugs, particularly for older patients, while others thought that their role should exclude drug dispensing, which they felt should be reserved to providers with medical training.

*No drugs [antihypertensives]… For the drugs I would prefer for it to remain at the hospital. When we take that up, what will be the responsibility of the doctor? When we get into drugs, they will fight us [[M: the healthcare providers]] yes… because we are interfering with their work. Everyone will have to play their role, I have screened, when she [community member] comes [to the health facility], she gets screened again, because it’s the work of the healthcare provider to screen her again and tell her it’s true what she [the CHP] has told you, you have it [hypertension].* (Community Health Promoter_A001)

CHPs also cited more direct benefits, with some reporting that they had hypertension themselves, and so the new knowledge would be useful for managing their own care.

#### Household production of health

Patient FGD data suggested that community members found CHPs to be key to improving health at the household level particularly for water, sanitation, and hygiene (WASH) activities and maternal child health. Community members and patients expressed gratitude for the work done by CHPs with several noting that CHPs should be appropriately remunerated for their work.

*Truly [CHPs] help, because they attend every meeting convened, they must talk about cleanliness in every meeting, about clinic [maternal child health] issues, they really help… we are grateful for CHPs, and they deserve to be paid but now they are being oppressed.* (Focus Group Discussion participant_A001)

Several CHPs reported using creative strategies to get individual community members to adopt healthy practices and follow health advice, and to manage their own workloads. These included maintaining an ‘open-door policy’ in CHPs’ homes and encouraging community members to reach out directly for urgent health matters. CHPs also noted that they were careful when they communicated health messages to people who used alternative medicines or sought faith healers. They highlighted the need to be respectful of people’s beliefs while encouraging use of the formal health system.

*When you ask them not to go for prayers, they won’t understand… so, you request them, because you must humble yourself, they are difficult. You say go [to the health facility] and confirm, you say you are fine, just go, get screened, when results come out well, I will also bring to you some [people] to take to the faith healer. You use wisdom to let them think you can bring other patients, but your goal is to convince them to seek care.* (Community Health Promoter_A001)

CHPs reported using their own resources to support community members who experienced difficulties in accessing care. For example, several CHPs said that they had paid for transportation to a health facility or bought food for patients to ensure they took their medication.

*‘Maybe she can say ‘I haven't eaten so I fear when I take the drugs, they might affect me’. So, when I have [money], I tell them go and buy milk and take the drugs or get some black coffee, I will buy you half a loaf of bread and then you take the drugs.’* (Community Health Promoter_B001)

#### Societal partnerships and community organizations

CHPs often drew on community leaders and other actors such as chiefs and village elders to support the expansion of community health programmes. Examples include using community meetings convened by local administrators to share health messages and reach a wide audience in a short time. Community leaders also often reinforced the health messages shared by CHPs. Further, CHPs were well-linked with other community actors such as the *nyumba kumi* (ten households) representatives who were a source of information concerning incidents that required CHP follow-up. One CHP noted how they worked with these representatives to stay abreast of health needs in the community.

*Yeah, if there is any challenge, I call them [household representatives] and they tell me as soon as it happens. I also have some women, who I have told if anything emerges, my phone number is here, I am always available, and I thank God, these people in my area, they listen to me. I don't switch off my phone, sometimes they even call me late at night. I know my area; I know it well. I get information.* (Community Health Promter_C001)

Several CHPs also held other leadership roles in the community. These roles not only enhanced their respect but also increased opportunities to follow up and share health messages with community members. One CHP noted:

*Mostly you wait until when they have the chamas [self-help group meetings]. The one I am in has men, but many of the people there are women. The good thing about women, if men are not there, the women will tell them what they were told. Sometimes, I see someone who I told to go to the clinic, and I ask them right there if they went. I talk to them about health issues… There are a lot of areas where you can plan and talk to people…* (Community Health Promoter_C002))

#### Perceived outcomes from greater CHP involvement in hypertension care

There were several benefits that different stakeholders thought were linked to greater CHP involvement in hypertension care. For example, patients noted individual-level benefits such as improved adherence to medication and early identification of BP problems before complications arose. Patients, CHPs, and healthcare providers also highlighted community and system-wide benefits such as increased community awareness of hypertension, better BP control, and reduced costs of care, particularly for older people, who often did not have reliable incomes. One patient who was hospitalized with hypertension-related complications noted:

*You know if I was present on a day in which CHWs were screening for hypertension, maybe I would not have reached this level. Maybe they would be there with medication, ‘so and so I am unwell do you have pressure drugs?’ Yes, I have them take. You understand me… that would be very simple… and when you are rushed from my home to the hospital, to and from is [Kshs] 600 [about USD 5]. You must have money. At the hospital, you also need money for medicines, so if they [CHPs] come and mobilize like that it will be better for us.* (Patient who defaulted treatment_002)

Healthcare providers and decision-makers cited CHPs’ roles in the adoption and maintenance of healthy lifestyles, a role that healthcare workers with professional training felt CHPs could effectively deliver because they were more embedded in the community.

*To me, it’s not a matter of accepting, it is a matter of appreciating the work i.e., done by the CHPs, the healthcare worker will not go to the house, or does not even live around the community members. But this CHP, lives or stays with the community members and they’ll visit them and come and tell the healthcare worker what is happening.* (Sub-county health manager_002).

### Unmet system requirements and constraints to CHP involvement in hypertension care

Our analysis also identified several factors that could constrain expansion of CHP roles to include hypertension care. These factors cut across health system and community dimensions: specifically, financing and supply chain concerns, tensions with frontline providers, and community scepticism towards CHP capacity.

#### Financing and commodity supply at community and health facilities

Several respondents, particularly decision-makers, attributed delays in roll-out of community-level hypertension services involving CHPs to practical implementation challenges.

*The screening services were initially supposed to start at the community level, the ‘how’ had been the challenge. Uh, who is going to support community health workers? Who is going to pay them? How are they going to screen for hypertension?* (National-level decisionmaker_001)

In October 2023, Kenya's President launched CHP kits that contained BP monitors and other supplies such as glucose strips and glucometers, fulfilling promises that CHPs would be empowered to offer non-communicable disease (NCD) services at the community level (MoH 2023). While the launch was widely welcomed, health managers raised concerns about continued financing and operational issues such as validation of the BP monitors, replacement of faulty equipment, and initial and refresher training of CHPs. Indeed, in the stakeholder workshops held some 9 months after the launch of the CHP kits, many county-level decision-makers noted that CHP-led hypertension screening had not commenced because of a lack of dedicated budget to purchase batteries required to operate BP monitors. Several participants pointed to inadequate planning and consultation between the national and county governments prior to the launch of CHP kits.

*Everybody’s asking today, okay, we have bought these kits, if they run out, who will buy them? Health [service delivery] is devolved [to counties]. But in the [county health] budget these [CHP kits] were not there. That’s what I’m saying. Both levels of government need to provide direction. Like now, who is to buy the batteries for the 3,650 BP machines? Here [county-level] we had not budgeted for it.* (County Health Manager_002)

County health managers were also concerned about sustainability, noting that county governments were expected to eventually cover CHP stipends and supplies for expanded CHP roles, yet these costs had not been factored into the 5-year county plan that informed annual health budgets. There was also the more immediate challenge of how training for CHPs would be funded. As noted above, CHPs had not received formal hypertension training at the time of data collection, and even after the kit launch, training was delayed for lack of provisions in the annual county health budget. Instead, health managers leveraged partnerships with NGOs, typically foundation-linked organizations and the corporate social responsibility arms of for-profit companies, to support CHP training and payment of stipends. Yet only a small number of organizations were able to offer this support, and where this was available it tended to be time-limited. This meant that CHP-led hypertension screening and linkage to care had to cease when NGO support ended.

Healthcare providers further raised concerns that emphasis on CHP-led hypertension screening risked directing resources to the community at the expense of health facilities. For example, they noted that while CHPs were equipped with BP machines, some PHC facilities lacked functioning equipment. Healthcare providers also noted that community screening for hypertension would result in higher demand for care at PHC facilities; yet it was unclear what efforts were being made to ensure adequate drug provision and staff training to manage the rising demand.

#### Community and facility health workforce

Despite the wide acceptance of CHP involvement in hypertension care, there were concerns around potential ‘role creep’, quality of care, and weak linkages between community and facility health workforce. Concerning role creep, one participant describing NGO-supported efforts to involve CHPs in hypertension screening at facility-level reported frontline providers’ initial resistance:

*That was a challenge at the beginning. When we recommended that we establish a desk for CHPs to screen for hypertension at health facilities, some [healthcare providers] said CHPs would be taking their responsibilities, but they need to look at the healthcare system as one unit so that they also understand the CHP has a role… So it is that argument that people need to have, and a change of perception on certain issues.* (NGO partner_001)

Decision-makers and healthcare providers also raised concerns about negative effects on quality of care if CHPs perceived themselves as ‘small doctors’. Health managers and providers were especially uncomfortable with extending CHPs’ roles to include delivery of hypertension medication, noting that healthcare providers could ‘lose’ the opportunity to monitor side effects, drug interactions, and medication errors, which were critical for high-quality patient care.

County health managers highlighted tensions between CHPs and health facility staff, particularly, secondary facilities (hospitals) which had fewer linkages with CHPs. These tensions resulted in a fragmented and siloed relationship. A county health manager explained:

*The role of the CHA is critical in bridging the gap [between communities and health facilities]. There is a problem with the link with the main hospitals. For example, the CHPs meet here with their CHA [supervisor] every month. But when you ask the management team, someone says, I see a group of people sit under that tree. What do they discuss? I don’t know. So, there’s a challenge. If, people are sitting in your compound every month, a group of people of like 20, 30, every month… under a tree for two hours, and then they disperse and there’s no interest in why they are there, there’s a problem… I think the problem is the orientation of the health system. I think it did not happen well.* (County health manager_004)

Frontline healthcare providers shared similar perspectives and described a lack of engagement and communication with CHPs at the hospital. For example, they were often not aware which CHPs were linked to their facilities, and how they could connect with the CHPs.

*Nobody talked to us, but I see the way it works is they have a note, a referral. They just pull the papers, they give the patient, it’s stamped, and the patient comes to the clinic. There’s no linkage. It should work together… And then the same thing with us in the clinic. If we have a patient who says, where I live the roads have been so bad, I haven’t gotten treatment for a week. So, if I knew my CHP, I’d just link the CHP to deliver the medicines… she can carry them and bring them to your house, she will take your blood pressure. But they [CHP] need to feel that they’re not working alone, they’re supported* (Healthcare worker_C002)

Relationships tended to be more positive at lower-level health facilities. Here facility-based staff were occasionally invited to attend the CHP monthly meetings to receive feedback about issues observed at household-level relevant to facility service delivery. However, several respondents noted that CHPs’ work was sometimes poorly valued by healthcare providers, especially those with low awareness about CHP roles, which, in turn, resulted in referrals from the community not being prioritized for care.

#### Community confidence in CHP capacity for hypertension care

Community members generally thought that CHP involvement in hypertension service delivery was beneficial as noted above, although some concerns were also raised. These related to CHPs’ ability to maintain confidentiality and their capacity to carry out hypertension-related tasks. These tasks were perceived to be more complex than WASH activities, or maternal and child health care that CHPs were known for. Like healthcare providers, several participants living with hypertension were concerned that CHPs might exceed their scope of practice and proposed that rather than equipping CHPs to provide hypertension services in the community, primary health care facilities should have adequate supplies for hypertension care tasks.

*So, these CHPs, they are like traditional birth attendants, the birth attendants are to provide first aid and accompany the woman to hospital. But now all the time, the traditional birth attendants do extra, even though they have been told if any emergency comes up, make a call, a vehicle will come here, but at times they try hard to show themselves [go beyond their role]. Now if we allow the CHPs to work on diabetes and hypertension, they will do more than their work. To help the community, these health centres and dispensaries should be equipped, that’s it, that’s the solution.* (Focus Group Discussion participant_C003)

To mitigate against these challenges, community members suggested that CHPs should receive additional training and be closely supervised, including being accompanied by healthcare providers.

*Listen, they may do, but how will they be trusted, the community here must know that they have gone for a training, and they are sent by the hospital to go and do the work there, that’s when they will be accepted. But not just even without the community knowing, they won’t be trusted. Yes…that there will be this and that, yeah, they should not just go to do the testing, some people will raise some concerns … they will be fought (Focus Group Discussion participant_C002)*

Participants also emphasised the need for community sensitization to raise awareness about CHPs’ expanded roles. In our FGDs, one participant highlighted how they refused to have their BP and sugar measured at their home because they were uncertain about the legitimacy of the CHP's visit.

## Discussion

Our study demonstrates strong community health system capacities that can be leveraged to support recently expanded roles for CHPs in Kenya that include hypertension care at community level. These include high coverage of community health units, motivated and creative CHPs, and partnerships and relationships that enable health production in communities. These capacities are further enhanced by increasing government commitment to professionalization of CHPs through training, supervision, provision of commodities, and payment of stipends. However, our study also highlights constraints that may affect sustainability and routinization of CHP roles in hypertension care.

We found that financing remains a major hurdle to implementing the expanded role of CHPs in hypertension care, a concern also raised elsewhere as the most common challenge facing national CHW programmes (Perry 2020, Masis et al. 2021). Mobilizing political will is thought to be a key factor for adequate financing of CHW programmes (USAID 2018). In Kenya, recent policy proposals point to the existence of political will, which has unlocked funding, with national and county governments partnering to pay CHP stipends. However, political will is often not sufficient if only used to garner support for the short-term. For example, the Village Health Guides programme in India was launched rapidly with significant political urgency, but with inadequate financing, and was eventually abandoned (Strodel and Perry 2019). In our study, county health managers thought that the CHP kits were hurriedly launched, and noted the budgetary strain of maintaining CHP kits, stipend payments, and meeting training costs, coupled with uncertainty around financial support by national government. These present significant threats to the sustainability of CHP roles in hypertension care.

Supply chain and commodity issues were another key challenge to the expansion of hypertension-care roles in CHW programmes identified in our study. These findings are consistent with evidence from multiple countries (Perry 2020, LeBan et al. 2021, Ludwick et al. 2022). Furthermore, CHP training costs and additional supplies required for NCD service delivery at community level had not been included in the county annual health budget and plans. Yet, we know from existing research that inadequate training can contribute to poorly prepared CHWs with a negative impact on motivation and commitment (Schleiff et al. 2021), with others noting that roll-out of CHW-led activities should include initial and refresher training as well as adequate supplies (Afzal et al. 2021). In decentralized health systems like our study context, consultation between national and sub-national governments is critical to support ownership and responsibility for tasks that will eventually be the responsibility of local decision-makers. Failure to secure ownership of subnational health managers with key roles in planning and budgeting for health resources can reduce the impact of national policies. Such sub-national managers are less likely to advocate for CHW expanded roles in hypertension care when caught between commodity stock-outs and training needs at community and facility-level and budget pressures, undermining the sustainability of the expanded CHP roles.

The linked challenges of financing and commodity supply highlight wider questions around how community health systems specifically, and the health sector more widely, are resourced. Kenya's public health system has been chronically underfunded (Masaba et al. 2020, Kairu et al. 2021), and additional allocations to support the CHP programme may not be possible without external donor funding or budget restructuring (Celia et al. 2017). However, research has shown that donor funding is often accompanied by fragmentation and inadequate co-ordination of health programmes (Afzal et al. 2021, Musoke et al. 2021). Further, recent shifts in global health funding (Callaway 2025, O'Sullivan and Puri 2025) underline the potential risk to sustainability of reliance on traditional donor funding avenues. In this study we found that CHP involvement in hypertension care commenced in a somewhat haphazard manner, predominantly led by NGOs with different approaches to training, hypertension service delivery, and incentive structures. As noted above, several of the NGOs supporting hypertension care in our study context were the corporate social-responsibility arms of for-profit companies, illustrating the emergence of new forms of funding support for health systems. Schneider et al. note that private–public partnerships are becoming increasingly common within health systems and particularly for CHW programmes (Schneider et al. 2022). Such public–private partnerships present an opportunity for resource mobilization for the health sector at national and sub-national levels but need to be carefully managed given possible divergent interests.

Expanding the role of CHPs to include hypertension care represents a significant organizational change. Such changes are often accompanied by perceived (or real) unequally distributed gains and losses for different actors and this can contribute to uneven buy-in as in our study. For example, healthcare providers were concerned about role boundaries, resource implications, and quality of care, while CHPs felt that an expanded role in hypertension care was consistent with their existing responsibilities, and an opportunity for enhanced legitimacy and respect. Studies from Cameroon, Mozambique, and Zambia report similar healthcare provider concerns around quality of care (Yakam and Gruénais 2009, Ferrinho et al. 2012) and increased workload (Jaskiewicz and Tulenko 2012). Our findings about community concerns are also consistent with study findings from Uganda and Western Kenya, which found that community members had low confidence in CHW capacity to deliver NCD (including hypertension) services (Rachlis et al. 2016, Musoke et al. 2021). Given that CHPs’ roles require both community and healthcare provider support, there is a need for careful engagement with patients, community members, and healthcare providers when expanding CHP roles. Healthcare providers may be more accepting of CHPs’ expanded tasks if roles are clearly defined and they are perceived to be a good fit in their setting (Glenton et al. 2021). Kenyan treatment guidelines and legislation define CHP roles as NCD screening and health education (MoH 2021, GoK 2023), but research suggests that CHW tasks for hypertension care could also include delivery of medication (Tsolekile et al. 2014, Jafar et al. 2020). For health professionals (and their associations) to support CHP expanded roles, clear communication including the rationale for change is necessary.

The existing positive relationships and goodwill between CHPs and community leaders reported in our study could be leveraged to facilitate support and acceptance and manage expectations about CHPs’ expanded roles in hypertension care. Several study respondents highlighted gaps in facility readiness for hypertension care, arguing for attention to the facility rather than at the community level. Thus, it would also be important to reinforce the message to both communities and healthcare providers that the expansion of CHP roles does not imply that facility strengthening should be neglected. The CHP programme relies on well-functioning health facilities and weak hypertension service delivery would undermine the expansion of CHP roles.

This study has demonstrated that to support the adoption and sustainability of expanded CHP roles, there is need for attention to the individual and organizational interactions across both community and health system sides. These ideas are supported by a recent review of community-centred interventions for chronic care that highlights the importance of attention to multi-level facilitators and barriers (Hassan et al. 2025) as well as a wider evidence base that recognises community health systems as relational, embedded within the community, and interfacing with the formal health system (Schneider and Lehmann 2016, LeBan et al. 2021, Schneider et al. 2022).

### Strengths and limitations

Our study focused on one county in Kenya, which limits the generalizability of our findings across the country. At the same time, this approach allowed for in-depth exploration of the study context, and our analysis has generated several insights that are potentially transferable to similar contexts. We used multiple data-collection methods and held stakeholder workshops for member-checking of our findings (Lincoln and Guba 1985), enhancing the trustworthiness of our findings. Our data did not explicitly speak to all the dimensions of Sacks et al.’s framework, such as information, learning, and accountability. This is in part because we applied the framework post hoc and did not integrate it into the data collection tools. Further, for the information, learning, and accountability dimension, given the fragmented nature of CHP involvement in hypertension care at the time of data collection, we had little evidence of any hypertension information entered in the community health information system, and how this was used to enhance learning and accountability for hypertension care. Nonetheless, we believe that our application of this framework has usefully extracted different elements across community and health systems dimensions for consideration when expanding CHW roles.

## Conclusion

This study illustrates the need for adopting a systems view in introducing changes to community health programmes. Such a view requires that interventions focus not just on the individual-level attributes of the CHW but consider community and health system relations, interactions, and organizational requirements that are key in shaping CHP roles and performance. Our findings point to the need for meaningful stakeholder engagement and openness to financing innovations going forward, as this may ultimately shape the sustainable uptake of CHP involvement in hypertension care in real-world settings. Our study may also inform those responsible for designing health policy in helping them anticipate implementation challenges that can be addressed in the design phase of similar policy interventions in community health systems. In 2012, the WHO encouraged piloting of interventions and rigorous assessment during the roll-out of new complex tasks for CHWs (WHO 2012). This recommendation remains relevant, particularly for hypertension services in which CHPs have not commonly been involved. The third aim of the IHCoR-Africa project is to contribute to this evidence gap in on-going research.

## Supplementary Material

czag020_Supplementary_Data

## Data Availability

The data underlying this article cannot be shared publicly to ensure the privacy of individuals that participated in the study. Data are however available from the authors upon reasonable request and with permission of the KEMRI Wellcome Trust Data Governance Committee.
