# Supplementary material for: Understanding what factors influence community health worker involvement in hypertension service delivery in Kenya: applying a community health system lens
Source: Health Policy Plan. 2026 Feb 24;41(4):627–39. doi: 10.1093/heapol/czag020 (PMC13089563; doi:10.1093/heapol/czag020)
Supplement: czag020_Supplementary_Data [file czag020_supplementary_data.docx]

**FGD Topic guide for patient/carer**

**Demographic data**

| Study ID* |  |
| --- | --- |
| Sub-county in Kilifi |  |
| Affiliated Health Facility |  |
| NCD condition |  |
| Duration of hypertension |  |
| Gender |  |
| Age |  |
| Marital status |  |
| Family carer (Yes/No) |  |
| Relationship to participant |  |

*Study ID to be allocated according to health facility type, interview type & participant number (i.e., Hosp1_FGD001)

**1. General perception of hypertension**

1.1 What does high blood pressure (hypertension) mean to you?

*Probes*

- *Why is it important to know about this condition?*
- *Do you have any symptoms and how would you describe these?*
- *What in your view has caused your hypertension?*

**2. First presentation**

2.1 Could you tell us about your experience about being diagnosed with high blood pressure. Do you remember when this was and how it was discovered?

***Probes***

- *Did you go see a healthcare worker because you had symptoms?*
- *Was it detected while you went to see a healthcare worker for something else?*
- *What facility did you go to? (e.g., dispensary/health centre, hospital, community outreach etc.)*
- *What type of healthcare worker did you see the first time that you were told that you have high blood pressure? (e.g., nurse, village/community health workers, doctor, etc.)*

2.2 Do you remember what you were told about high blood pressure?

*Probes*

- *How did you feel about your diagnosis?*
- *What questions did you have? what were you worried about?*
- *Were your questions answered in a way that you could understand?*
- *How did you feel about the way you were diagnosed?*

**3. Follow-up appointments**

3.1 Could you tell us what happened after that first time when you were told you had high blood pressure?

*Probes*

- *What were you advised to do?*
- *Were you given an appointment to come back later for another check-up?*
- *Was the appointment in the same facility or a different place?*
- *Who saw you for the appointment?*
- *Do you remember what you talked about in that appointment?*
- *How easy do you find it to attend medical appointments? How so?*

3.2 Could you tell us what happened after the first appointment?

*Probes*

- *Did you have to come back again for other appointments? Why?*
- *Were your appointments all in the same facility or different facilities?*
- *How often did you have to come back?*
- *What happened during these appointments?*
- *Who did you see, what did you discuss, what examination / tests did you undergo?*
- *Who initiated medical appointments, was the health facility or yourself? How was this arranged?*

**4. Treatment**

*4.1 Could you tell us what you were told about how your high blood pressure was going to be treated?*

*Probes*

- *Were you given any medications?*
- *Who was it that prescribed your medication?*
- *How often do you see a healthcare worker about your medication?*
- *Where do you get your medications from?*
- *How easy do you find it to get your medications? (For example, distance to dispensary, financial ability to buy medicine, etc.)*

4.2 Are you using other remedies to treat your high blood pressure? If yes, what are you using and why?

*Probes*

- *Where do you usually get such remedies?*
- *How often do you resort to them?*

**5. Management and control**

5.1 How do you manage your high blood pressure, day-to-day? And what do you find easy or difficult about managing your blood pressure?

*Probes*

- *What do you do to keep your high blood pressure under control?*
- *Do you monitor your blood pressure regularly? If so, how?*
- *Do you have measurement device at home, or do you go to a healthcare facility? Etc.*
- *Do you sometimes skip taking your medication, and if so, why? (e.g., organising or storing medicines; having to take more than one at regular intervals; need to adjust daily activities to align with medication schedule; side effects)*

5.2 How easy do you find it to get your medications for high blood pressure?

*Probe*

- *Where do you get the medication from?*
- *How easy do you find it to take your medications regularly?*
- *Have you or are you experiencing any difficulties in paying for your medications? If yes, in what sense and how frequently?*

5.3 Have you made any changes to your diet in order to manage your high blood pressure? If so, what have you changed and how easy do you find this?

*Probes*

- *How easy do you find it making these changes?*
- *What are the greatest challenges to maintaining these changes?*

5.4 Are you doing any physical activities or exercises to help manage your high pressure? If so, what activities are you doing and how easy do you find this?

5.5 What support do you get from your family or the community to help you manage your high blood pressure? Are you part of any patient support group?

*Probes*

- *Emotional support?*
- *Financial support (e.g., paying for medications)*
- *Practical support (e.g., information; transport to clinics)*

5.6 Where do you look for information or advice on how to manage your high blood pressure and how useful has this been?

*Probe*

- *Do you get your information, advice, or support from healthcare providers or from other sources? Could you say a bit more about that?*

**6. Complications of hypertension**

6.1 Have you experienced any other health problems related to your high blood pressure since you were diagnosed? How was this managed?

*Probes*

- *What health problem/s did you experience?*
- *How was it/where these discovered?*
- *Who did you see for the treatment of this problem? what type of facility did you go to? what type of healthcare worker did you see there?*

**7. Experiences of interacting with the health service**

7.1 How easy is it for you to go and see a nurse or doctor about your high blood pressure when you need to?

*Probes*

- *Are you able to see someone when you need to?*
- *How easy do you find it to attend medical appointments? (For example, geographical distance to health facilities, waiting time, financial issues, transportation, etc.)*

7.2 Thinking about everything that has happened to you since you have been first diagnosed with high blood pressure: Is there anything that could have been done by the doctor or nurse to make your experience better?

*Probes*

- *How easy/difficult is it to understand the information given to you.*
- *How easy/difficult is it to ask questions.*

7.3 What types of support would you like to have to make it easier for you to manage your high blood pressure?

7.4 Is there anything that could be better about the current treatment or support you received?

**8. Village/Community health workers**

8.1 What do you think about the work that is done by village/community health workers in your community? Tell us more about this.

Probes:

- *For what condition/s do they care? How do they provide this care?*
- *Do you think they are important in delivering healthcare? Do you think they are effective?*
- *Do you trust their knowledge and skills to provide care?*

8.2 Do you interact with community health workers for the management of your hypertension?

If yes: [Interviewer: assure and emphasise again that the interviews are confidential and anonymous and that they can express their views about CHW safely]

Probes:

- *What kind of care do you receive from them for hypertension?*
- *How often do you see them?*
- *Do you trust community health care workers to give you the care that you need for your high blood pressure? Do you think they are helpful and effective?*
- *Do you think they have the knowledge and skills to give you the care that you need?*

*If no:*

- *Why do you think CHW are not involved in the care of your hypertension?*

8.3 [If patient does not receive care from CHW for hypertension] - How would you feel about village/community health workers getting involved in managing your high blood pressure?

*Probes*

- *Would you trust them to give you information and advice on how to manage your high blood pressure?*
- *How would you feel if they supplied you with your medication for high blood pressure?*
- *Would you trust them to do regular blood pressure check-ups with you?*
- *Do you think they could do more to help detect high blood pressure in your community, and if so, what should they do?*
- *Would you be okay with them visiting you at home?*

**9. Close**

9.1 Is there anything else about your experiences of interacting with health care services and how well they support you in managing your high blood pressure that you would like to add?

**IDI Topic guide for patient/carer**

*1. Please introduce yourself to the participant and ask them to introduce themselves*

*2. Please ensure that* ***the participant has read (or has had read to them) the study information sheet****, outlining the aims of the study, how their data will be used, and potential risks and benefits of participating, and* ***has signed the informed consent form*** *for participation.*

*3. Please confirm that the participant is happy to have their interview audio-recorded and that they know they can stop the interview at any point or refuse to answer any of the questions.*

**Demographic data**

| Study ID* |  |
| --- | --- |
| Sub-county in Kilifi |  |
| Affiliated Health Facility |  |
| NCD condition |  |
| Duration of hypertension |  |
| Family carer (Yes/No) |  |
| Relationship to participant |  |

**1. General perception of hypertension**

1.1 What does high blood pressure (hypertension) mean to you?

*Probes*

- *Why is it important to know about this condition?*
- *Do you have any symptoms and how would you describe these?*
- *What in your view has caused your hypertension?*

**2. First presentation**

2.1 Could you tell us about your experience about being diagnosed with high blood pressure. Do you remember when this was and how it was discovered?

***Probes***

- *Did you go see a healthcare worker because you had symptoms?*
- *Was it detected while you went to see a healthcare worker for something else?*
- *What facility did you go to? (e.g., dispensary/health centre, hospital, community outreach etc.)*
- *What type of healthcare worker did you see the first time that you were told that you have high blood pressure? (e.g., nurse, village/community health workers, doctor, etc.)*

2.2 Do you remember what you were told about high blood pressure?

*Probes*

- *How did you feel about your diagnosis?*
- *What questions did you have? what were you worried about?*
- *Were your questions answered in a way that you could understand?*
- *How did you feel about the way you were diagnosed?*

**3. Follow-up appointments**

3.1 Could you tell us what happened after that first time when you were told you had high blood pressure?

*Probes*

- *What were you advised to do?*
- *Were you given an appointment to come back later for another check-up?*
- *Was the appointment in the same facility or a different place?*
- *Who saw you for the appointment?*
- *Do you remember what you talked about in that appointment?*
- *How easy do you find it to attend medical appointments? How so?*

3.2 Could you tell us what happened after the first appointment?

*Probes*

- *Did you have to come back again for other appointments? Why?*
- *Were your appointments all in the same facility or different facilities?*
- *How often did you have to come back?*
- *What happened during these appointments?*
- *Who did you see, what did you discuss, what examination / tests did you undergo?*
- *Who initiated medical appointments, was the health facility or yourself? How was this arranged?*

**4. Treatment**

*4.1 Could you tell us what you were told about how your high blood pressure was going to be treated?*

*Probes*

- *Were you given any medications?*
- *Who was it that prescribed your medication?*
- *How often do you see a healthcare worker about your medication?*
- *Where do you get your medications from?*
- *How easy do you find it to get your medications? (For example, distance to dispensary, financial ability to buy medicine, etc.)*

4.2 Are you using other remedies to treat your high blood pressure? If yes, what are you using and why?

*Probes*

- *Where do you usually get such remedies?*
- *How often do you resort to them?*

**5. Management and control**

5.1 How do you manage your high blood pressure, day-to-day? And what do you find easy or difficult about managing your blood pressure?

*Probes*

- *What do you do to keep your high blood pressure under control?*
- *Do you monitor your blood pressure regularly? If so, how?*
- *Do you have measurement device at home, or do you go to a healthcare facility? Etc.*
- *Do you sometimes skip taking your medication, and if so, why? (e.g., organising or storing medicines; having to take more than one at regular intervals; need to adjust daily activities to align with medication schedule; side effects)*

5.2 How easy do you find it to get your medications for high blood pressure?

*Probe*

- *Where do you get the medication from?*
- *How easy do you find it to take your medications regularly?*
- *Have you or are you experiencing any difficulties in paying for your medications? If yes, in what sense and how frequently?*

5.3 Have you made any changes to your diet in order to manage your high blood pressure? If so, what have you changed and how easy do you find this?

*Probes*

- *How easy do you find it making these changes?*
- *What are the greatest challenges to maintaining these changes?*

5.4 Are you doing any physical activities or exercises to help manage your high pressure? If so, what activities are you doing and how easy do you find this?

5.5 What support do you get from your family or the community to help you manage your high blood pressure? Are you part of any patient support group?

*Probes*

- *Emotional support?*
- *Financial support (e.g., paying for medications)*
- *Practical support (e.g., information; transport to clinics)*

5.6 Where do you look for information or advice on how to manage your high blood pressure and how useful has this been?

*Probe*

- *Do you get your information, advice, or support from healthcare providers or from other sources? Could you say a bit more about that?*

**6. Complications of hypertension**

6.1 Have you experienced any other health problems related to your high blood pressure since you were diagnosed? How was this managed?

*Probes*

- *What health problem/s did you experience?*
- *How was it/where these discovered?*
- *Who did you see for the treatment of this problem? what type of facility did you go to? what type of healthcare worker did you see there?*

**7. Experiences of interacting with the health service**

7.1 How easy is it for you to go and see a nurse or doctor about your high blood pressure when you need to?

*Probes*

- *Are you able to see someone when you need to?*
- *How easy do you find it to attend medical appointments? (For example, geographical distance to health facilities, waiting time, financial issues, transportation, etc.)*

7.2 Thinking about everything that has happened to you since you have been first diagnosed with high blood pressure: Is there anything that could have been done by the doctor or nurse to make your experience better?

*Probes*

- *How easy/difficult is it to understand the information given to you.*
- *How easy/difficult is it to ask questions.*

7.3 What types of support would you like to have to make it easier for you to manage your high blood pressure?

7.4 Is there anything that could be better about the current treatment or support you received?

**8. Village/Community health workers**

8.1 What do you think about the work that is done by village/community health workers in your community? Tell us more about this.

Probes:

- *For what condition/s do they care? How do they provide this care?*
- *Do you think they are important in delivering healthcare? Do you think they are effective?*
- *Do you trust their knowledge and skills to provide care?*

8.2 Do you interact with community health workers for the management of your hypertension?

If yes: [Interviewer: assure and emphasise again that the interviews are confidential and anonymous and that they can express their views about CHW safely]

Probes:

- *What kind of care do you receive from them for hypertension?*
- *How often do you see them?*
- *Do you trust community health care workers to give you the care that you need for your high blood pressure? Do you think they are helpful and effective?*
- *Do you think they have the knowledge and skills to give you the care that you need?*

*If no:*

- *Why do you think CHW are not involved in the care of your hypertension?*

8.3 [If patient does not receive care from CHW for hypertension] - How would you feel about village/community health workers getting involved in managing your high blood pressure?

*Probes*

- *Would you trust them to give you information and advice on how to manage your high blood pressure?*
- *How would you feel if they supplied you with your medication for high blood pressure?*
- *Would you trust them to do regular blood pressure check-ups with you?*
- *Do you think they could do more to help detect high blood pressure in your community, and if so, what should they do?*
- *Would you be okay with them visiting you at home?*

**9. Close**

9.1 Is there anything else about your experiences of interacting with health care services and how well they support you in managing your high blood pressure that you would like to add?

**IDI Topic Guide for Village Health Workers / Community Health Volunteers**

**Introduction**

- Give an introduction. Explain what the interview aims to achieve and what you would like to discuss with the respondent (i.e., a range of questions to clarify their roles, the context in which they work, and the potential for them to participate in hypertension diagnosis, care and management)
- Confirm participant has read the study information sheet
- Confirm participant has signed the consent form
- Do you have any questions before we start the interview?
- Thank participants for agreeing to the interview, and ask permission to switch on the recorder

**Please fill out the following:**

VHC/CHV role/tasks (If more than one, describe main tasks/roles): ________________________

Years in role: ___________________________________________________________

Local community/village: _________________________________________________________

Years living in community/village: _________________________________________________________

**1. General role description**

1.1 What motivated you to become a Village Health Worker/Community Health Volunteer?

1.2 Can you tell me about your work? What are your main tasks as a Village Health Worker/Community Health Volunteer?

*Probes*

- *providing health advice: what type of advice, on what and where (villagers’ homes)?*
- *treating patients: for what types of conditions/ailments?*
- *referring patients: for what reasons and to what facility/ies*
- *how much time (per week) do you spend on tasks such as health advice, treating and referring patients?*
- *do your see your role as mainly being prevention or treatment?*

1.2 How confident do you feel in performing these tasks?

*Probes*

- *are there tasks that you feel more/less confident in? Why is that?*
- *are there any tasks that you would like to offer but you are unable to? Why is that?*
- *are there any tasks that you feel you should not be performing? Why is that?*

1.3 What has your training been like, do you feel it has prepared you well for your role?

*Probes*

- *Who provided the training and in what?*
- *How often is the training provided?*

1.4 Do you receive any financial compensation for your work as a Village Health Worker / Community Health Volunteer?

*Probes*

- *type and frequency of financial compensation (e.g., weekly, monthly, quarterly stipend)*
- *who provides financial compensation?*
- *do you feel the compensation is appropriate given the tasks that you are performing? Why is that?*

1.5 How well do you think is your work accepted?

*Probes*

- *by members of the community?*
- *by other healthcare workers?*
- *by the (local/regional/national) government?*

1.6 How would you describe your relationship with healthcare workers such as nurses, doctors, and others?

*Probes*

- *do you see yourself as a member of the healthcare workforce?*
- *do you feel that healthcare workers consider Village Health Workers/Community Health Volunteers as members of the healthcare workforce?*
- *what could be done to make you feel more integrated into health system?*

**2. Current role in hypertension diagnosis, treatment, and management**

2.1 How would you describe your understanding of hypertension?

*Probes*

- *Why is it important to know about this condition?*
- *What in your view are the causes of hypertension?*
- *Do you think that hypertension is a problem in your village/community/country? If yes, why do you think so?*
- *Do you know people in your village/community who have hypertension?*

2.2 Do you look after people with hypertension as part of your role?

*Probes*

- *If yes, what are your specific tasks?*
  - *How confident do you feel performing these tasks?*
  - *Have you received any specific training to perform these tasks?*

***(Skip if already answered in 2.2)***

2.3 Are you currently or have in the past measured the blood pressure of other people?

*Probes*

- *If yes, how confident did you feel performing this task?*
- *Have you received any specific training to perform this task?*

***(Skip if already answered in 2.2)***

2.4 If you know someone in your village/community who has hypertension and has problems because of this, what would you do?

**3. Potential future role in hypertension diagnosis, treatment, and management**

3.1 How do you feel about the role of Village/Community Health Workers in providing care to hypertension patients? Do you think they should be involved?

*Probe*

*[if a positive response]*

- *Why do you think this is not happening already?*
- *What do you see their role could be?*
- *How do you think this would benefit healthcare workers in facilities and patients?*

*[If negative response]*

- *Why do you think they should not be involved?*
- *What could be changed for their involvement to be appropriate?*

## Topic guide for healthcare worker IDI

Please fill out the following:

Health worker role (If more than one, capture all roles): ________________________

Type of facility: _________________________________________________________

***Key informant profile***

1. Can you tell me a little about your work? What is your role in healthcare delivery? And how long have you been in this role?
2. What is your role in the diagnosis and/or treatment of hypertension?

***Organizational profile and policy***

1. Are you familiar with any formal guidelines, protocols or standard operating procedures for the diagnosis and/or treatment of hypertension? If so, what are these?

Probes:

- *If familiar with a guideline/protocol/SOP:*
  - *Do you use it in your daily practice?*
  - *If yes, how easy do you find it to work according to the guideline/protocol/SOP?*
    - *What are the main challenges of working according to the guideline/protocol/SOP in your daily practice?*
  - *If you do not use the guideline/protocol/SOP, what are the reasons for this?*
  - *Could we have a copy? When were they last updated? How do you access them?*

***Hypertension diagnosis***

1. What is the role of this facility in the diagnosis and management of hypertension?
2. Could you describe how someone with high blood pressure is typically diagnosed as a hypertensive patient?

*Probes*

- *How is blood pressure measured for a hypertension diagnosis? How many times is it measured?*
- *If a patient is presenting to the healthcare service – How does this presentation occur?*
- ***If through screening – Probe further****:*
  - *How is screening carried out? (e.g., location, by whom, for whom)*
  - *Are there regular or frequent outreach activities?*
  - *How are these organised and who delivers these?*

1. How is the responsibility for hypertension management structured within this facility? How well does this work? Why do you think so?

Probes:

- Is there a team at your facility that specifically manages hypertension? Is there a team leader?
- How well does this teamwork? Why?

**Appointments after diagnosis (what happens after the initial diagnosis)**

1. Could you describe what happens after a patient is found to have high blood pressure?

*Probes*

- Are they given a follow up appointment?
- *Who initiates medical appointments, the health facility, or the patient? How is this arranged?*
- *Which facilities do they attend for the follow-up appointment?*
- *What happens during these appointments? Who do they see, what do they discuss, what examination / tests do they undergo?*
- *How often do patients attend appointments?*
- *What happens if a patient misses an appointment?*

1. What do you think are some of the challenges encountered in the follow-up appointments of hypertension patients?
2. Are there specific arrangements for patients to see the same health worker or team of health workers over time? How well do these arrangements work? Why?

**Complications**

1. How are complications of hypertension detected and monitored?
2. Could you tell us when and how a patient is referred for specialised care?

*Probes*

- *Which facilities are patients referred to for specialised care?*
- *What would the reason for referral to a specialist be?*
- *How is this patient referred?*
- *How often does a hypertensive patient see a specialist?*

1. How easy is it to get an appointment with a specialist?
2. What do you think are some of the challenges encountered in the referral process of patients from primary care to specialists?

**Referral pathways**

1. Do you receive feedback when you refer patients? Do you give feedback when patients are referred to you? How well do these mechanisms work? Why?

**Medication**

1. When and how is hypertension medication prescribed to a patient with high blood pressure?

*Probes*

- *Who prescribes hypertension medication?*

1. How is patient adherence to medication monitored?
2. How is non-adherence to medication detected and corrected?
3. What challenges have you encountered, if any, in the management / treatment of hypertension?

**Information and support to patients**

1. What information, advice, or support is given to patients on how to manage their hypertension?

*Probes*

- *On taking medication?*
- *On monitoring blood pressure?*
- *On physical activity or exercise?*
- *On food or dietary changes?*

1. Do you offer additional services to your patients during clinic visits? Which are these services?

***Professional support for healthcare workers***

1. Do you / did you receive any formal training for the diagnosis or management of hypertension? If so, what did the training involve?

*Probes*

- *Do you feel that the training has equipped you sufficiently to perform the tasks you are meant to perform? If not, why not?*
- *What other training do you think you need? Why do you think that?*

1. What other support have/are you receiving to enable you to diagnose/treat hypertension?

Probes

- *Do you have regular meetings with other health workers who manage hypertension within this facility? or from neighbouring facilities that are the same level/type as your facility?*
- *Do you have mechanisms to regularly access medical specialists to seek advice? How well do these mechanisms work? Why?*

***Community healthcare workers***

1. To what degree are community health care workers currently involved in the diagnosis and treatment of hypertension patients?

- If they are involved, could you provide more detail on what they do and how you work with them?
- *What are the main benefits of having CHW involved? (for the community, for healthcare workers)*
- *What are the main challenges from your perspective?*
- If CHW are not currently involved, what do you think their role could be?

*[if a positive response] probe further*

- *Why do you think that CHW are not already involved?*
- *What precise tasks do you think CHW could take on?*
- *What training do you think they would need and who should provide it?*
- *How do you see yourself working with CHW to deliver hypertension diagnosis and/or treatment*
- *What do to think are the main challenges of getting CHW more closely involved?*

*[If negative response]*

- *Why do you think CHW should not be involved?*
- *What do you think needs to be in place so that CHW can take on a more formal role in delivering hypertension diagnosis and/or treatment?*

***Views on current pathways and improvements***

1. From your perspective, what would an ideal service for the diagnosis and management of hypertension look like? What would need to be in place for this to be realised?

- *Probe for organisational, human resource, financing and regulatory requirements*
